# Redox Properties of *Bacillus subtilis* Ferredoxin:NADP^+^ Oxidoreductase: Potentiometric Characteristics and Reactions with Pro-Oxidant Xenobiotics

**DOI:** 10.3390/ijms25105373

**Published:** 2024-05-14

**Authors:** Mindaugas Lesanavičius, Daisuke Seo, Gintarė Maurutytė, Narimantas Čėnas

**Affiliations:** 1 Department of Xenobiotics Biochemistry, Institute of Biochemistry, Life Sciences Center, Vilnius University, Saulėtekio Av. 7, LT-10257 Vilnius, Lithuania; mindaugas.lesanavicius@gmc.vu.lt (M.L.); gintare.maurutyte@gmail.com (G.M.); 2 Division of Material Sciences, Graduate School of Natural Science and Technology, Kanazawa University, Kakuma, Kanazawa 920-1192, Japan; dseo@se.kanazawa-u.ac.jp

**Keywords:** quinones, nitroaromatics, flavins, redox cycling, ferredoxin:NADP^+^ oxidoreductase, single-electron reduction

## Abstract

*Bacillus subtilis* ferredoxin:NADP^+^ oxidoreductase (*Bs*FNR) is a thioredoxin reductase-type FNR whose redox properties and reactivity with nonphysiological electron acceptors have been scarcely characterized. On the basis of redox reactions with 3-acetylpyridine adenine dinucleotide phosphate, the two-electron reduction midpoint potential of the flavin adenine dinucleotide (FAD) cofactor was estimated to be −0.240 V. Photoreduction using 5-deazaflavin mononucleotide (5-deazaFMN) as a photosensitizer revealed that the difference in the redox potentials between the first and second single-electron transfer steps was 0.024 V. We examined the mechanisms of the reduction of several different groups of non-physiological electron acceptors catalyzed by *Bs*FNR. The reactivity of quinones and aromatic *N*-oxides toward *Bs*FNR increased when increasing their single-electron reduction midpoint redox potentials. The reactivity of nitroaromatic compounds was lower due to their lower electron self-exchange rate, but it exhibited the same trend. A mixed single- and two-electron reduction reaction was characteristic of quinones, whereas reactions involving nitroaromatics proceeded exclusively via the one-electron reduction reaction. The oxidation of FADH^•^ to FAD is the rate-limiting step during the oxidation of fully reduced FAD. The calculated electron transfer distances in the reaction with nitroaromatics were close to those of other FNRs including the plant-type enzymes, thus demonstrating their similar active site accessibility to low-molecular-weight oxidants despite the fundamental differences in their structures.

## 1. Introduction

Ferredoxin:NADP^+^ oxidoreductases (FNRs, EC 1.18.1.2) are ubiquitous flavoenzymes found in all domains of life and belong to the class of dehydrogenase–electrontransferases. In oxygenic photosynthesis, monomeric plant-type FNR with a molecular mass of 35–36 kDa transfers a redox equivalent from reduced iron–sulfur protein ferredoxin (Fd) to NADP^+^, providing NADPH for CO_2_ assimilation [1,2,3,4,5]. In non-photosynthetic processes, FNR catalyzes the reduction of Fd at the expense of NADPH supplying reduced Fd for nitrate assimilation (roots), biosynthesis of isoprenoids (the malaria parasite *Plasmodium falciparum*), and so on [5,6,7]. Under iron-limiting conditions, the FMN-containing low-potential electron carriers flavodoxins (Flds) function as the physiological redox partners of FNR in place of Fd [8,9]. However, stronger and more specific interactions occur between FNR and Fd in comparison to those occurring with Fld [10].

**Figure 1:**
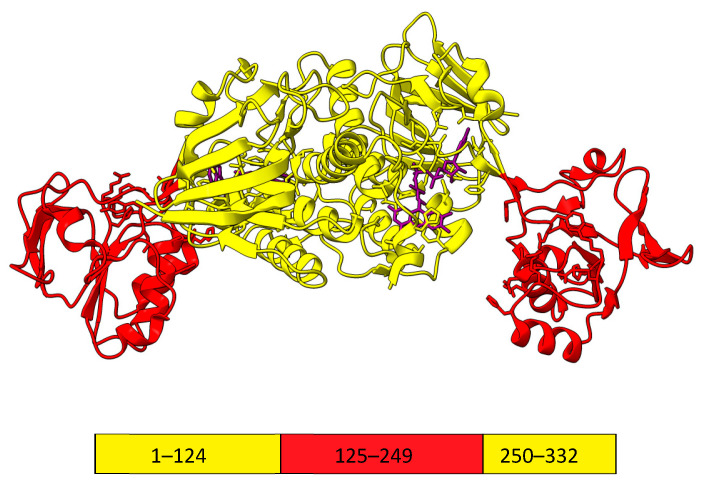
The 3D structure of a *Bs*FNR dimer (PDB id: 3LZX). FAD-binding domain is colored yellow, while the NADP(H)-binding domain is colored red. The box denotes amino acids comprising each domain in a monomer. The bound FAD is shown in purple. The molecular graphics were made using UCSF ChimeraX [14].

FNR from the Gram-positive bacterium *Bacillus subtilis* (*Bs*FNR) belongs to a novel group of FNRs classified as NADPH-thioredoxin reductase (TrxR)-type FNR based on their high sequence similarity with bacterial TrxRs [3,11]. A feature that distinguishes *B. subtilis*, as well as other representatives of the TrxR-type FNR, FNRs from *Rhodopseudomonas palustris*, and *Chlorobaculum tepidum*, from plant-type FNR is their homodimeric structures (Figure 1) [12,13]. Another distinguishing feature is that the FAD-binding domain comprises two discontinuous segments separated by the segment of the NADPH-binding domain (Figure 1), while in plant-type FNRs, each domain is composed of a single peptide segment [3]. In *Bs*FNR, the FAD-binding domain is composed of residues 1–124 and 250–332, and the NADP(H)-binding domain is composed of residues 125–249. The FAD-binding and NADP(H)-binding domains are connected by two flexible hinge regions that enable the rotation of the domains relative to one another. This is plausible since, in the case of *Bs*FNR, the distance between bound NADP^+^ and the isoalloxazine ring of FAD is ca. 15 Å in the crystal structure, which is too distant for an efficient hydride transfer. The crystal structure of *Bs*FNR shows two aromatic amino acids, Tyr50 and His324, located over the *si* and *re* faces of the isoalloxazine ring of FAD, respectively. These amino acids are almost parallel to the isoalloxazine ring, with the corresponding distance being 3.3–3.6 Å, and form a π-π stacked structure [13].

The mechanism of the catalysis of *Bs*FNR with its physiological substrates has been partially characterized. At pH 7.0 and 10 °C, NADPH reduces the enzyme, with *k* ≥ 500 s^−1^, while its reoxidation with NADP^+^ occurs much more slowly and partially [15]. The reactions are accompanied by the transient formation of charge transfer complexes of NADP^+^/H with reduced/oxidized FAD, respectively, which absorb at longer wavelengths. The maximal steady-state turnover rates of *Bs*FNR using the non-physiological electron acceptor ferricyanide or Fe_4_S_4_-containing *B. subtilis* ferredoxin (*Bs*Fd) are close to 1000 s^−1^ and 50–100 s^−1^ based on single-electron transfer, respectively [16,17]. In the reduction of *Bs*FNR with reduced *Bs*Fd, the rate-limiting step is the reduction of the FAD semiquinone (FADH^•^) intermediate, with *k* > 50 s^−1^ [15]. A possible physiological function of *Bs*FNR is the reduction of *Bs*Fd and flavodoxins, which further act as electron donors for cytochrome P450 BioI [18,19], lipid desaturase [20], and NO synthase, which, in this case, consists only of the oxygenase domain [21].

There are other, virtually untouched aspects of *Bs*FNR redox reactions. In particular, *B. subtilis*, as a soil bacterium, is naturally exposed to redox-active humic compounds, quinones generated by various fungi and bacteria, and nitroaromatic industrial compounds [22,23,24,25]. Like other FNRs, *Bs*FNR is a potential target of these compounds, and it can stimulate their toxic effects by reducing them to free radicals that cause oxidative stress, or, alternatively, it can participate in their reductive detoxification [25,26]. Another aspect is the possible action of *Bs*FNR as a target of redox-cycling drugs, analogous to what was suggested for the malaria parasite *Plasmodium falciparum* FNR [27]. Although *B. subtilis* is generally harmless to humans, separate cases of it being pathogenic have been reported [28,29]. On the other hand, TrxR-type FNRs have been found in most firmicutes, including pathogens, and, for example, *Bs*FNR has 70% amino acid sequence homology with TrxR-like FNR from pathogenic *Listeria monocytogenes* [30]. For these reasons, we employed steady- and pre-steady-state kinetics methods to determine the structure–activity relationships of *Bs*FNR in reactions with a series of redox-cycling quinones, nitroaromatic compounds, and aromatic *N*-oxides. For the quantitative analysis of the obtained data and comparison with other FNRs, the redox potentials of *Bs*FNR were also determined.

## 2. Results

### 2.1. The Determination of Redox Potentials of BsFNR

The potentiometric properties of TrxR-type FNRs are largely unexplored, with the exception of the previously determined standard redox potential ($E_{7}^{0}$, the potential of the E-FAD/E-FADH^−^ redox couple) and corresponding single-electron transfer potentials (E-FAD/E-FADH^•^ and E-FADH^•^/E-FADH redox couples) of *R. palustris* FNR [31]. The $E_{7}^{0}$ of flavoenzymes can be determined using the Haldane relationship, according to which the ratio of the bimolecular rate constants of forward and reverse reactions yields the equilibrium constant of the reaction (*K*) [31,32]. This, in turn, is related to the difference in the standard redox potential of the reactants ($∆E^{0}(V)=0.0295\times \log K$ for a two-electron transfer). Because the estimation of the kinetic parameters of the reverse reaction, the reduction of NADP^+^ by *Bs*FNR, is complicated by *Bs*Fd substrate inhibition [15], we examined the enzyme reactions using the NADP(H) analogue 3-acetylpyridine adenine dinucleotide phosphate (APADP(H), $E_{7}^{0}$ = −0.258 V [33]). In this case, *K* is expressed as the ratio of bimolecular rate constants ($k_{\mathrm{cat}}/K_{m}$) of APADPH oxidation and APADP^+^ reduction. In the forward reaction, APADPH (50–500 µM) was generated in situ using the glucose-6-phosphate/glucose-6-phosphate dehydrogenase reduction system, and the reaction rate was monitored following the reduction of 1.0 mM of ferricyanide. The reaction rates did not depend on ferricyanide concentration in the range of 0.25–1.0 mM. The reaction was characterized by $k_{\mathrm{cat}}^{\mathrm{app}}$ = 105.0 ± 3.2 s^−1^ and $k_{\mathrm{cat}}/K_{m}$= 1.5 ± 0.2 × 10^6^ M^−1^s^−1^ (on a two-electron basis). The enzymatic reduction of APADP^+^ (50–500 µM) by 200 µM of NADPH proceeded with $k_{\mathrm{cat}}^{\mathrm{app}}$ = 35.9 ± 1.0 s^−1^ and $k_{\mathrm{cat}}/K_{m}$= 3.6 ± 0.4 × 10^5^ M^−1^s^−1^. Thus, according to calculations *K* = 4.1 ± 0.7, and $E_{7}^{0}$ for the enzyme was −0.240 ± 0.002 V.

**Figure 2:**
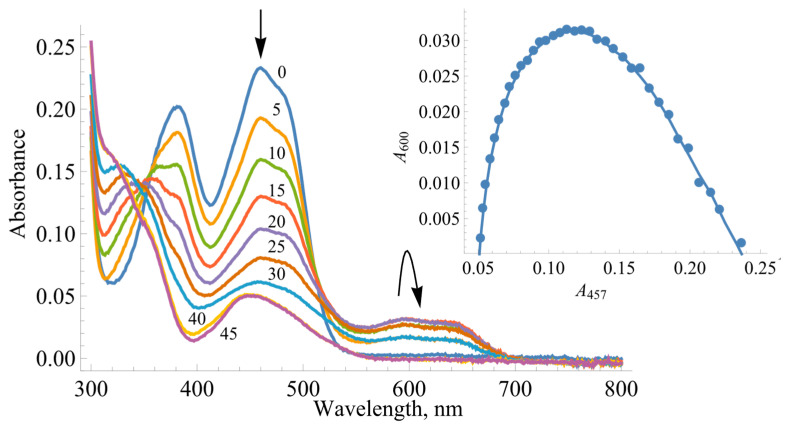
The determination of the redox potentials of *Bs*FNR. The photoreduction of 20 µM of *Bs*FNR at different times of illumination, with the arrows indicating the changes in the spectra. The numbers next to the curves correspond to the total duration of illumination in minutes. Inset shows the interdependence of absorbance changes at 457 and 600 nm during the photoreduction of 20 µM of *Bs*FNR.

During the photoreduction of *Bs*FNR with 5-deazaFMN and EDTA as a photosensitizer, a neutral (blue) FAD semiquinone (FADH^•^) transiently formed, as evidenced by the characteristic broad absorption band in the 550–650 nm range (Figure 2). The extinction coefficient ε_600_ for *Bs*FNR FAD is not known; however, the amount of E-FADH^•^ can be assessed from the A_457_ vs. A_600_ plot (Figure 2 inset) using the value ε_600_ = 3.6 mM^−1^cm^−1^ for the Y50G *Bs*FNR mutant [34]. In this case, the maximal amount of semiquinone stabilized was 44%. The separation between the two single-electron-transfer potentials ($∆E_{7}^{1}=E_{7}^{{E-\mathrm{FAD}/E-\mathrm{FADH}}^{•}}-E_{7}^{{{E-\mathrm{FADH}}^{•}/E-\mathrm{FADH}}^{-}}$) can be calculated based on the semiquinone formation constant *K*_s_ (Equations (1) and (2)):

(1) $$\frac{{\left[{E-\mathrm{FADH}}^{•}\right]}_{\max}}{{\left[E-\mathrm{FAD}\right]}_{\mathrm{tot}}}=\frac{\sqrt{K_{s}}}{2+\sqrt{K_{s}}}$$

(2) $$\Delta E_{7}^{1}\left(V\right)=0.059\times \log K_{s}$$

where ${\left[{E-\mathrm{FADH}}^{•}\right]}_{\max}$ is the maximal concentration of semiquinone and ${\left[E-\mathrm{FAD}\right]}_{\mathrm{tot}}$ is the total concentration of enzyme. The calculations yield *K*_s_ = 2.572 and $∆E_{7}^{1}$ = 0.024 V, and, subsequently, $E_{7}^{{E-\mathrm{FAD}/E-\mathrm{FADH}}^{•}}$ = −0.228 V and $E_{7}^{{{E-\mathrm{FADH}}^{•}/E-\mathrm{FADH}}^{-}}$ = −0.252 V.

### 2.2. Steady-State Kinetics and Oxidant Substrate Specificity of BsFNR

**Figure 3:**
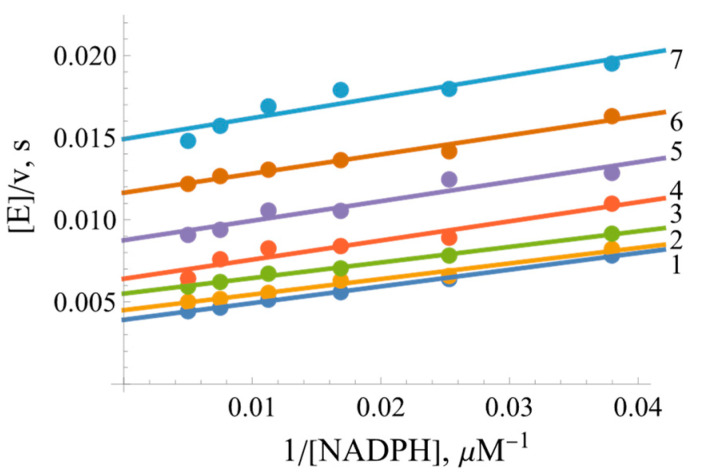
Lineweaver–Burk plots of the steady-state kinetics of the oxidation of NADPH catalyzed by *Bs*FNR with varied concentrations of juglone (5-hydroxy-1,4-naphthoquinone): 1—200 µM of juglone, 2—133 µM of juglone, 3—89 µM of juglone, 4—59 µM of juglone, 5—39 µM of juglone, 6—26 µM of juglone, and 7—17 µM of juglone.

Earlier studies performed on FNRs from other sources indicate that juglone (5-hydroxy-1,4-naphthoquinone) and its derivatives are potent nonphysiological oxidants of these enzymes [31,35]. A series of parallel lines were obtained in double reciprocal plots upon varying the concentration of NADPH with a constant concentration of juglone (Figure 3) and vice versa. This shows that the *Bs*FNR-catalyzed quinone reductase reaction proceeds via a “ping-pong” mechanism.

As calculated according to Equation (9) (presented in the Materials and Methods Section), the *k*_cat_ value for juglone reduction at an infinite NADPH concentration is equal to 360.2 ± 9.8 s^−1^, and the values of the bimolecular rate constants ($k_{\mathrm{cat}}/K_{m}$) for juglone and NADPH are equal to 4.35 ± 0.23 × 10^6^ M^−1^s^−1^ and 1.00 ± 0.07 × 10^7^ M^−1^s^−1^, respectively.

**Figure 4:**
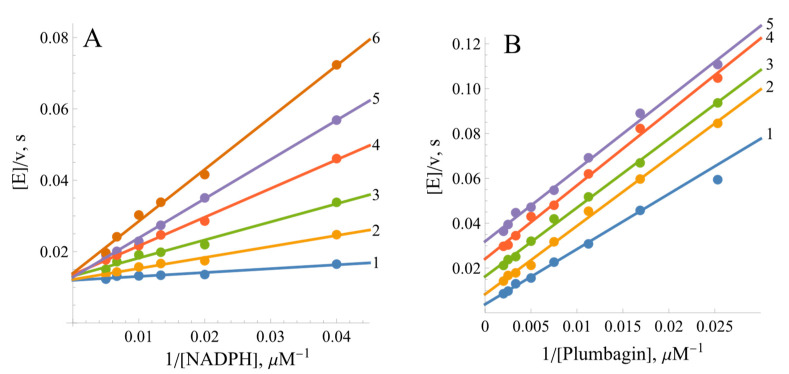
Inhibition of BsFNR-catalyzed reactions by the reaction product NADP^+^. (**A**) Competitive inhibition at varied concentrations of NADPH in the presence of 200 µM of plumbagin (5-hydroxy-2-methyl-1,4-naphthoquinone): 1—no NADP^+^, 2—100 µM of NADP^+^, 3—250 µM of NADP^+^, 4—500 µM of NADP^+^, 5—750 µM of NADP^+^, and 6—1000 µM of NADP^+^. (**B**) Uncompetitive inhibition at varied plumbagin concentrations in the presence of 100 µM of NADPH: 1—no NADP^+^, 2—500 µM of NADP^+^, 3—1000 µM of NADP^+^, 4—2000 µM of NADP^+^, and 5—3000 µM of NADP^+^.

The quinone reductase reaction of *Bs*FNR is inhibited by the reaction product NADP^+^. At a fixed oxidant concentration, NADP^+^ acts as a competitive inhibitor towards NADPH, increasing the slopes of the Lineweaver–Burk plots but not affecting the maximal rate of reaction (Figure 4A). Its inhibition constant (*K*_i_), calculated according to Equation (10) (Materials and Methods), is equal to 62.6 ± 10.5 µM. In turn, at a fixed NADPH concentration, NADP^+^ acts as an uncompetitive inhibitor towards the oxidant, decreasing the maximal reaction rate but not increasing the slopes of the Lineweaver–Burk plots (Figure 4B). Its uncompetitive *K*_i_, calculated according to Equation (11) (shown in the Materials and Methods Section), is equal to 261 ± 27 µM.

A series of quinones (Q), aromatic nitrocompounds (ArNO_2_), and aromatic *N*-oxides (ArN→O) with single-electron reduction potentials ($E_{7}^{1}$) from 0.09 to −0.494 V were studied to determine the specificity of *Bs*FNR towards oxidizing substrates. The latter group of compounds, derivatives of 3-amino-1,2,4-benzotriazine-1,4-dioxide (tirapazamine), were studied in connection with their antimicrobial activity [36]. The single-electron acceptors ferricyanide, benzyl viologen, and FeEDTA^−^ were also studied. Among the studied forms of ArNO_2_, there are explosives, such as tetryl and 2,4,6-trinitrotoluene; antibacterial agents, like nitrofurantoin and nifuroxime; and the anticancer agent CB-1954. The apparent maximal reduction rate constants $k_{\mathrm{cat}}^{\mathrm{app}}$ of the electron acceptors at 100 µM of NADPH and their respective $k_{\mathrm{cat}}/K_{m}$ values are given in Table 1.

**Figure 5:**
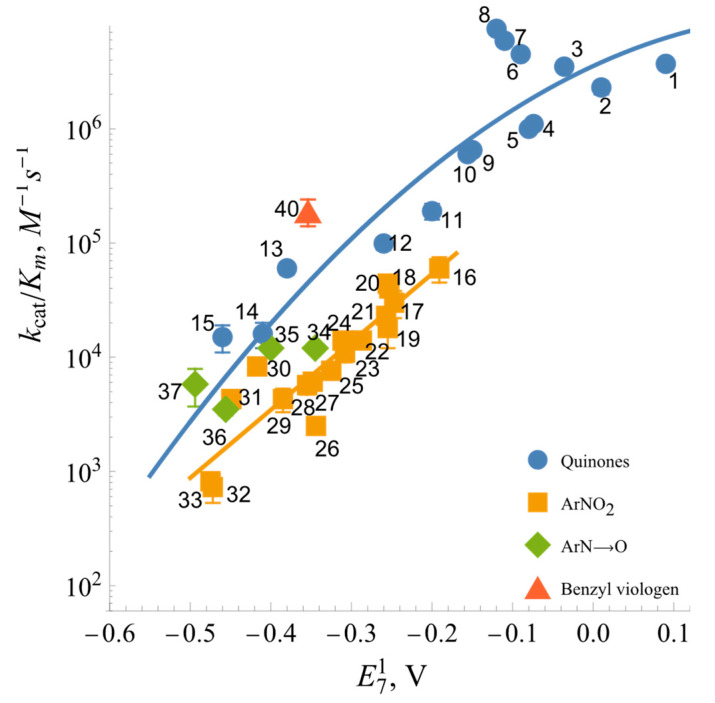
Dependence of the reactivity of quinones, nitroaromatic compounds, aromatic *N*-oxides, and benzyl viologen on their single-electron reduction midpoint potentials (log_10_ scale). Numbers and reduction potentials of the compounds are given in Table 1.

The log $k_{\mathrm{cat}}/K_{m}$ values for ArNO_2_ exhibit a linear dependence on their $E_{7}^{1}$ values (Table 1, Figure 5), with tetryl (16) being the most potent electron acceptor in that group. On the other hand, the log $k_{\mathrm{cat}}/K_{m}$ values for quinones with a similar $E_{7}^{1}$ are about an order of magnitude higher. Moreover, quinones and aromatic *N*-oxides together exhibit a distinct parabolic dependence on their $E_{7}^{1}$ values (Figure 5). One should also note the single-electron acceptor benzyl viologen (40), with its steady-state kinetic constants being close to those of quinones with similar $E_{7}^{1}$ values.

We found that *Bs*FNR reduces Q and ArNO_2_ to their radicals. Typically, the single-electron flux for quinone reduction by NAD(P)H-oxidizing enzymes is defined as the ratio of the rate of 1,4-benzoquinone-mediated reduction of the added cytochrome *c* to the double rate of 1,4-benzoquinone-mediated NAD(P)H enzymatic oxidation at pH < 7.2 [41]. Such an approach is based on the fast reduction of cytochrome *c* by the 1,4-benzosemiquinone (*k* ≈ 10^6^ M^−1^s^−1^) and its slow reduction by the hydroquinone form. In the case of the *Bs*FNR-catalyzed reduction of 20–100 µM of 1,4-benzoquinone by 100 µM of NADPH, the single-electron flux was equal to 17%. On the other hand, during the reduction of 9,10-phenanthrenequinone, the calculated single-electron flux was equal to 70%, with superoxide dismutase (SOD) (100 U/mL) inhibiting cytochrome *c* reduction by 43%. The assessment of the single-electron flux for the reduction of ArNO_2_ is also based on the ArNO_2_^•−^-mediated reduction of added cytochrome *c*. In the presence of 50 µM of NADPH and 100 µM of TNT or *p*-nitrobenzaldehyde, the rate of *Bs*FNR-catalyzed reduction of 50 µM of cytochrome *c* was equal to 180–200% of the NADPH oxidation rate, corresponding to 90–100% single-electron flux. SOD inhibited the reactions by 10–30%, reflecting the rapid reoxidation of the radicals with O_2_ and the participation of the superoxide in the reduction of cytochrome *c*.

**Figure 6:**
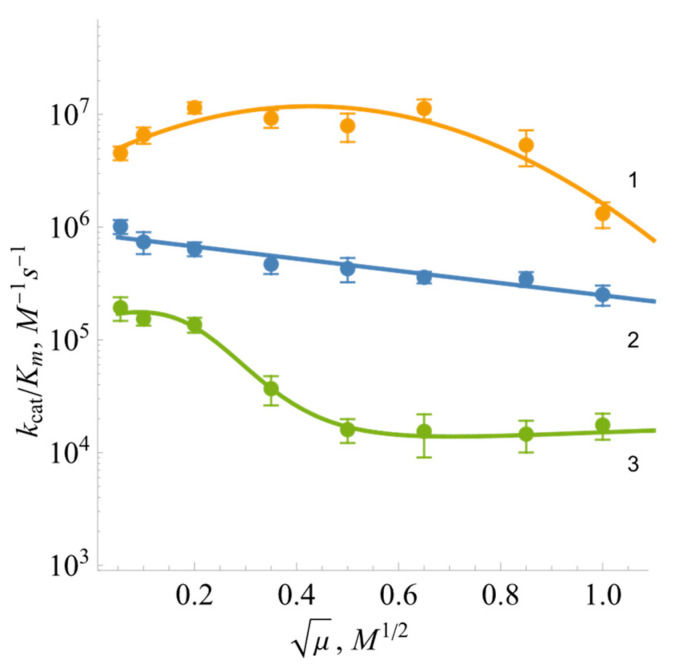
The dependence of log *k*_cat_/*K_m_* of *Bs*FNR-catalyzed reaction on the ionic strength of the buffer: 1—potassium ferricyanide, 2—1,4-naphthoquinone, and 3—benzyl viologen.

In order to characterize the role of electrostatic interactions in the reduction of oxidants, experiments were performed, with varying ionic strengths, using negatively charged oxidant ferricyanide, positively charged benzyl viologen, and neutral 1,4-naphthoquinone (Figure 6). The log $k_{\mathrm{cat}}/K_{m}$ for ferricyanide exhibits a parabolic character with a peak value at I ≈ 0.25 M. The reactivity of uncharged 1,4-napthoquinone decreases slightly upon increasing ionic strength. In general, the log $k_{\mathrm{cat}}/K_{m}$ of benzyl viologen decreased upon increasing the ionic strength of the buffer solution (Figure 6).

### 2.3. Pre-Steady-State Kinetics of BsFNR Oxidation under Multiple Turnover Conditions

**Figure 7:**
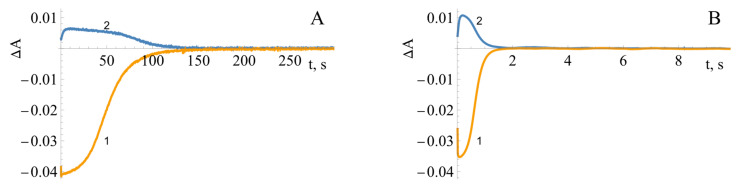
The absorbance changes at 460 nm (1, orange) and 600 nm (2, blue) during the reduction of *Bs*FNR (5 µM) by 50 µM of NADPH and its subsequent reoxidation by O_2_ (**A**) or 250 µM of duroquinone (**B**) (concentrations were reported after mixing).

Some insight into the reoxidation mechanism of *Bs*FNR can be gleaned from the spectral changes of the enzyme-bound FAD during the multiple turnovers under aerobic conditions in the presence of NADPH and tetramethyl-1,4-benzoquinone (duroquinone, DQ). DQ does not absorb above 460 nm, and its semiquinone form is rapidly reoxidized by oxygen [37]. The control experiment performed in the absence of DQ showed an initial fast phase of FAD reduction by NADPH, which was then followed by a much slower reoxidation by O_2_ seen at 460 nm and a transient increase in absorbance at 600 nm (Figure 7A), at the same time scale. The addition of DQ enhanced the reoxidation rate by up to two orders of magnitude (Figure 7B).

**Figure 8:**
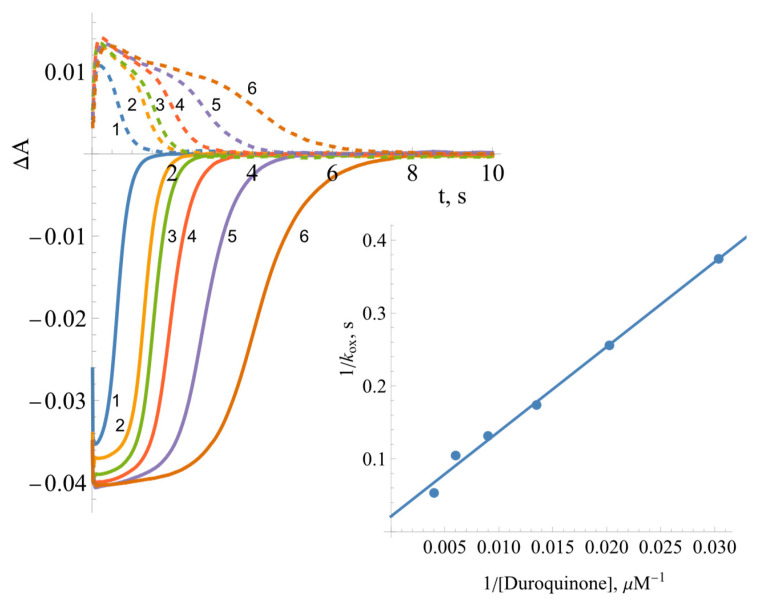
The kinetics of *Bs*FNR (5 µM) reduction and reoxidation under multiple turnover conditions with a varying concentration of duroquinone in the presence of 50 µM of NADPH followed at 460 nm (solid curves) and 600 nm (dashed curves): 1—250 µM of duroquinone, 2—166 µM of duroquinone, 3—111 µM of duroquinone, 4—74 µM of duroquinone, 5—49 µM of duroquinone, and 6—33 µM of duroquinone (all concentrations were reported after mixing). The inset shows the Lineweaver—Burk plot for the dependence of the apparent first-order rate constant of the reoxidation of reduced FAD on the concentration of duroquinone.

The reaction rate constants were calculated according to Equation (3) [42]:

(3) $$k_{\mathrm{ox}}=\frac{{\left[\mathrm{NADPH}\right]}_{0}}{{\left[E_{\mathrm{red}}\right]}_{\max}\times t_{1/2(\mathrm{off})}}$$

where [NADPH]_0_ is the initial concentration of NADPH, [E_red_]_max_ is the maximal concentration of the reduced enzyme formed during the turnover, and t_1/2(off)_ is the time interval between the formation of the half-maximum amount of E_red_ and its decay to the half-maximum value. According to the absorbance difference between the oxidized and reduced enzyme forms, that is, Δε_460_ = 9.02 mM^−1^cm^−1^ [15], the maximal extent of enzyme reduction is 91% (Figure 7A). This yields *k*_ox_ = 0.22 s^−1^ for the reoxidation of *Bs*FNR by O_2_, a value that is similar to the enzyme NADPH oxidase activity, 0.17 s^−1^. Analogous calculations at various DQ concentrations (Figure 8) yield the value of *k*_ox(max_._)_ = 48.3 ± 6.7 s^−1^ (Figure 8 inset), which may reflect the maximum rate of the oxidative half-reaction.

**Figure 9:**
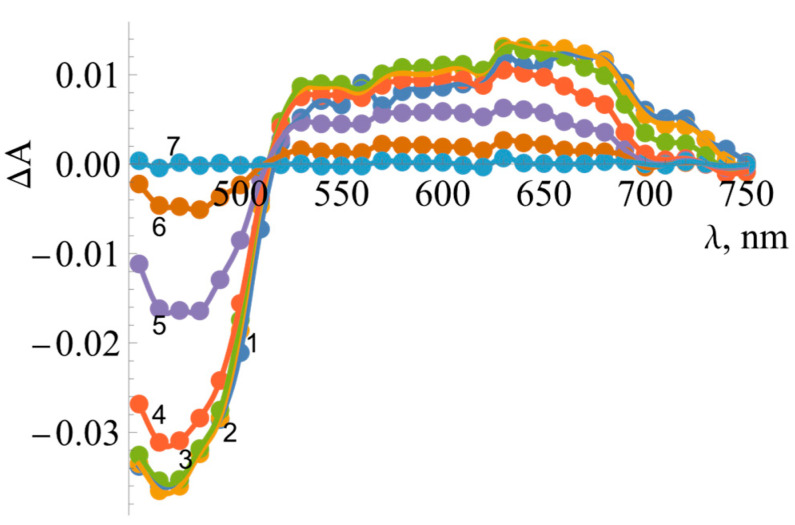
The spectra of reaction intermediates formed during the turnover of 5 µM of *Bs*FNR in the presence of 50 µM of NADPH and 250 µM of duroquinone (concentrations were reported after mixing). Differences in absorbance are shown at several timepoints over the 450–750 nm range. Spectra correspond to 50 ms (1, blue), 100 ms (2, orange), 250 ms (3, green), 500 ms (4, red), 750 ms (5, purple), 1 s (6, brown), and 5 s (7, teal) after mixing.

Measurements taken at different wavelengths showed that the intermediate product of reoxidation by DQ has a flat absorption maximum between 600 and 700 nm (Figure 9). The decay of the reaction intermediate begins at about 500 ms after the start of the reaction.

## 3. Discussion

**Figure 10:**
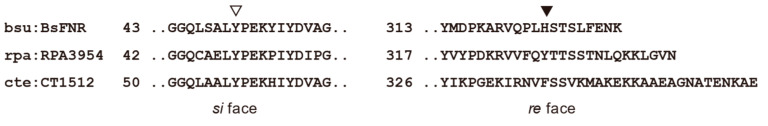
The partially aligned amino acid sequences of *Bs*FNR, *R. palustris* FNR (RPA3954) and *Chlorobaculum tepidum* FNR (CT1512). The residues stacked on the *si*-face (Tyr50 in *Bs*FNR) and *re*-face (His324 in *Bs*FNR) of the isoalloxazine ring portion are indicated with arrowheads. The alignment was performed using ClustalW [47].

Since TrxR-type FNRs are relatively poorly studied enzymes, our studies focused on revealing similarities and differences between the redox properties of *Bs*FNR and other groups of FNRs. For the first time, we determined the standard redox potential of *Bs*FNR, i.e., −0.240 V, which is significantly more positive than the $E_{7}^{0}$ of FNRs from other sources, e.g., −0.280 V (*P. falciparum* [43]) or −0.342 V (spinach [44]). Most importantly, however, it is sufficiently more positive than the potential of its TrxR-type homologue, FNR from *R. palustris*, with a value of −0.276 V [31]. The amino acids surrounding the isoalloxazine ring of FAD in both enzymes are largely conserved, except for the change of Tyr328 to His324 and Thr329 to Ser325 in *Bs*FNR (Figure 10) [12,13]. The possible effect of the latter substitution on *Bs*FNR $E_{7}^{0}$ is not clear, but the effect of the presence of His324 is similar to the case for flavodoxin from *Desulfovibrio vulgaris*_,_ where Tyr98His substitution increased the $E_{7}^{0}$ of FMN by 0.07 V [45]. It is believed that the presence of the imidazole group of histidine stabilizes the anionic form of the reduced flavin, i.e., makes its oxidation more difficult. This elevated $E_{7}^{0}$ value agrees with previous data on the inefficient reoxidation of *Bs*FNR by NADP^+^ [15] and the possible role of this enzyme in Fd/Fld reduction at the expense of NADPH. On the other hand, the FAD semiquinone stability of *Bs*FNR, 44% at equilibrium (Figure 2), is slightly but not drastically higher than that of *R. palustris* FNR, i.e., 26.5% [31]. The stability of FADH^•^ may be due to the presence of Tyr50, Asp57, Ile296, and Thr326, the last of which forms a H-bond with N5 of isoalloxazine, in the active site of *Bs*FNR, corresponding to the Tyr49, Asp56, Ile 300, and Thr330 of *R. palustris* FNR [46]. The semiquinone-stabilizing effect of the aforementioned amino acid residues has been discussed in our previous work [31].

Due to the possible involvement of *Bs*FNR in reactions with redox-active agents in the soil, further attention was paid to the analysis of reactions of model redox-active compounds of different groups. Here, we found a series of features common to a wide range of FNRs: (i) the “ping-pong” mechanism of reaction and the way of NADP^+^ inhibition (Figure 3 and Figure 4) are similar to those of other FNRs [31,35]. This result shows that the reductive and oxidative half-reactions occur separately but at the same binding site. The considerable variation in the $k_{\mathrm{cat}}^{\mathrm{app}}$ values of the reactions with different oxidants (Table 1) suggest that oxidative half-reaction is the rate-limiting step. The competitive inhibition of NADP^+^ with respect to NADPH and the uncompetitive inhibition with respect to the oxidant is explained by the specific case of the “ping-pong” mechanism, wherein NADP^+^ strongly competes with NADPH for binding to the oxidized enzyme but binds poorly to the reduced form of the enzyme and/or does not reoxidize it [48]. (ii) Another feature common to various classes of FNRs is the dependence of log $k_{\mathrm{cat}}/K_{m}$ of various classes of oxidants on their $E_{7}^{1}$, i.e., the absence of a pronounced impact of their structural features on reactivity (Figure 5) [31,35]. This may correspond to the “outer sphere” single-electron transfer model [49]. In this case, the rate constant for the electron transfer between reactants (*k*_12_) depends on their electron self-exchange rate constants (*k*_11_ and *k*_22_) and the equilibrium constant of reaction (*K*):

(4) $$\log K=\frac{∆E^{1}\left(V\right)}{0.059}$$

(5) $$k_{12}=\sqrt{k_{11}\times k_{22}\times K\times f}$$

and *f* is expressed as

(6) $$\log f=\frac{\log^{2}\;K}{4\log\left(\frac{k_{11}\times k_{22}}{Z^{2}}\right)}$$

where *Z* is the frequency factor, 10^11^ M^−1^s^−1^. According to Equations (5) and (6) for the reaction between an electron donor and a series of homologous electron acceptors with similar *k*_22_ values, log *k*_12_ will exhibit a parabolic (or linear, in the case of low exothermicity) dependence on Δ*E*^1^. We observed this experimentally: the *k*_22_ of ArNO_2_, ≈ 10^6^ M^−1^s^−1^, is 100 times lower than that of Q and ArN→O, ≈ 10^8^ M^−1^s^−1^ [40,50], leading to a 10-fold lower reactivity of ArNO_2_ when compared to quinones of similar $E_{7}^{1}$ values (Figure 5). (iii) The transient formation of 500–700 nm absorbing species during enzyme reoxidation is typical for other FNRs [31,35] and consistent with a two-step reoxidation scheme, namely, FADH^−^→FADH^•^→FAD, with FADH^•^ oxidation being the rate-limiting step. This means that the obtained $k_{\mathrm{cat}}/K_{m}$ of the compounds (Table 1) reflect FADH^•^ oxidation. The small absorption increase above 700 nm is uncharacteristic of FADH^•^ and indicative of a parallel formation of other reaction intermediates, e.g., the complexes of FADH^•^ with NADP(H) observed in adrenodoxin reductase [51,52,53,54].

In parallel, some differences from previously studied FNRs were also observed. The experiments conducted at varied ionic strengths may characterize the region of *Bs*FNR that interacts with charged oxidants. The data on *Anabaena* PCC7119 and *P. falciparum* FNRs demonstrate the bell-shaped dependences of log $k_{\mathrm{cat}}/K_{m}$ on ionic strength irrespective of the charge of the oxidants [35]. This can be attributed to an interaction with both the negatively charged glutamate and positively charged lysine residues, which are located close to the dimethylbenzene part of the isoalloxazine ring [55,56,57]. However, the data in Figure 6 show that the oxidants interact with the negatively charged amino acid residue(s) of *Bs*FNR. In this case, the potential candidates are Asp285 and, possibly, Asp57 near the isoalloxazine ring of FAD [13]. Most likely, the parallel conformational changes in the protein occur at high ionic strength, decreasing the reactivity of the uncharged 1,4-naphthoquinone (Figure 6). The positively charged fragment of the *Bs*FNR FAD domain, Lys264, Lys317, andArg319 [13], which possibly electrostatically interacts with the negatively charged *Bs*Fd [18], may not interact with non-physiological electron acceptors because it is too far from the isoalloxazine ring [15]. However, the most unexpected difference from other FNRs was the mixed one- and two-electron reduction of quinones by *Bs*FNR. Both *Anabaena* PCC7119 and *P. falciparum* FNRs reduce both quinones and ArNO_2_ only in a single-electron fashion [35,58]. The fact that the Glu301Ala mutant of *Anabaena* FNR reduced 1,4-benzoquinone in a 50% single-electron manner can be explained by its significant destabilization of FADH^•^, 8% at equilibrium [58]. However, *Bs*FNR reduces 1,4-benzoquinone with only 17% of single-electron flux, although the FADH^•^ stabilization is 44% (Figure 2). Similarly, another TrxR-type *R. palustris* FNR with 26.5% FADH^•^ stabilization reduces 1,4-benzoquinone with 54% single-electron flux [31]. Since this regularity was observed in the case of two TrxR-type FNRs, it is possible that single- and two-electron reduction flux are not determined by FADH^•^ stability alone but also by the specificity of the isoalloxazine surroundings. One of the operative factors could be the unusual π-π stacking between the isoalloxazine ring and the aromatic Tyr50 and His324 groups [16,34]. This feature, including its possible influence on the degradation of xenobiotics, is the subject of our further research.

Finally, the influence of *Bs*FNR structural peculiarities in reactions with redox-active xenobiotics can be quantitatively described. According to Mauk et al., the electron transfer distance in metalloproteins reacting with inorganic complexes under infinite ionic strength (i.e., no electrostatic effects) can be related to the metalloproteins’ electron self-exchange rate constant (*k*_11_) (Equation (7)) [59]:

(7) $$R_{p}\left(Å\right)=6.3-0.35\ln k_{11}$$

This approach has been used to estimate the *R*_p_ values in the single-electron oxidation of various flavoenzyme dehydrogenase-electrontransferases [31,35]. However, the values thus obtained should be regarded cautiously due to the possibility of electron transfer distances being overestimated since, in the case of dehydrogenase-electrontransferases, the dimethylbenzene part of the isoalloxazine ring is partly exposed to the solvent. Thus, these data can only be used to compare the “intrinsic” reactivity of different FNRs.

Since there is some uncertainty regarding the mechanism of quinone reduction by *Bs*FNR, we will only analyze the case of ArNO_2_ reduction. At $∆E_{7}^{1}=0$, $k_{12}=\sqrt{k_{11}\times k_{22}}\;$(Equation (5)). At $E_{7}^{1}$ of ArNO_2_ = −0.228 V, i.e., the potential of E-FADH^•^/E-FAD redox couple, and considering that the *k*_22_ of ArNO_2_ is equal to 10^6^ M^−1^s^−1^ [50], the value of log *k*_11_ calculated according to the data in Figure 5 is equal to 3.11 ± 0.15. According to Equation (7), this yields *R*_p_ = 3.8 ± 0.1 Å. For comparison, *R*_p_ values calculated in an analogous way for nitroreductase reactions are equal to 4.4 Å (*Anabaena* FNR), 4.9–5.6 Å (*P. falciparum* FNR), and 5.4 ± 0.2 Å (*R. palustris* FNR) [31,35]. This shows that these *R*_p_s are close to each other, including for the plant-type FNRs. Complementing the *R. palustris* FNR data [31], this shows that the active sites of TrxR-type FNRs are not characterized by decreased accessibility to low-molecular-weight oxidants, which may be caused by the shielding of the isoalloxazine ring by the flexible C-terminal region, or by the domain movement of the protein.

In addition to providing fundamental information on the structure and catalysis of this class of FNRs, these data may also have some practical implications, e.g., with respect to using the FNRs of this group in bioelectrocatalysis [60]. In this case, the environment of the redox cofactor and the distance from the surface of the protein usually determine the rate of the corresponding electrochemical reactions.

## 4. Materials and Methods

### 4.1. Reagents and Enzymes

*B. subtilis* ferredoxin:NADP^+^ oxidoreductase was prepared as previously described, and its concentration was determined spectrophotometrically according to ε_457_ = 12.3 mM^−1^cm^−1^ [16]. NADP(H), 3-acetylpyridineadenine dinucleotide phosphate (APADP^+^), horse heart cytochrome *c*, superoxide dismutase, glucose oxidase, catalase, glucose 6-phosphate, glucose-6-phosphate dehydrogenase from *Leuconostoc mesenteroides*, 5-deaza-FMN, and other commercially available reagents were obtained from Sigma-Aldrich (St. Louis, MO, USA) and used as received.

### 4.2. Steady-State Kinetics

The steady-state kinetics experiments were performed using a Cary60 UV/Vis spectrophotometer (Agilent Technologies, Santa Clara, CA, USA). All experiments were performed in 0.02 M Hepes/NaOH + 1 mM EDTA buffer solution, with a pH of 7.0, at 25 °C. The kinetic data were fitted to the Michaelis–Menten equation in Mathematica (Wolfram Research, Inc., Mathematica, Version 14.0, Champaign, IL, USA (2024)) (Equation (8)) to yield the steady-state parameters of the reactions, namely, the catalytic constants $k_{\mathrm{cat}}^{\mathrm{app}}$ and bimolecular reaction rate constants (or catalytic efficiency constants) $k_{\mathrm{cat}}/K_{m}$ of the oxidants under a fixed concentration of NADPH:

(8) $$\frac{v}{\left[E\right]}=\frac{k_{\mathrm{cat}}^{\mathrm{app}}\left[S\right]}{K_{m}+\left[S\right]}$$

where v is the reaction rate, [E] is the concentration of *Bs*FNR, [S] is the concentration of the oxidant, and $k_{\mathrm{cat}}^{\mathrm{app}}$ represents the number of molecules of NADPH oxidized by a single native molecule of the enzyme per second at saturated concentrations of both substrates. The fitted parameters are equal to the reciprocal intercepts and slopes of Lineweaver–Burk plots, [E]/v vs. 1/[S], respectively. The concentrations of the enzyme used in these experiments were 5–50 nM. The kinetic parameters of the steady-state reactions according to the “ping-pong” mechanism were calculated according to Equation (9):

(9) $$\frac{v}{\left[E\right]}=\frac{k_{\mathrm{cat}}^{\mathrm{app}}\left[Q\right]\left[\mathrm{NADPH}\right]}{K_{m}^{\mathrm{NADPH}}\left[Q\right]+K_{m}^{Q}\left[\mathrm{NADPH}\right]+\left[\mathrm{NADPH}\right]\left[Q\right]}$$

The competitive inhibition constant *K*_i_ of NADP^+^ vs. NADPH was calculated according to Equation (10):

(10) $$\frac{v}{\left[E\right]}=\frac{k_{\mathrm{cat}}^{\mathrm{app}}\left[\mathrm{NADPH}\right]}{K_{m}^{\mathrm{NADPH}}\left(1+\frac{\left[{\mathrm{NADP}}^{+}\right]}{K_{i}}\right)+\left[\mathrm{NADPH}\right]}$$

The uncompetitive inhibition constant *K*_i_ of NADP^+^ vs. electron acceptor (Q) was calculated according to Equation (11):

(11) $$\frac{v}{\left[E\right]}=\frac{k_{\mathrm{cat}}^{\mathrm{app}}\left[Q\right]}{K_{m}^{Q}+\left[Q\right]\left(1+\frac{\left[{\mathrm{NADP}}^{+}\right]}{K_{i}}\right)}$$

The rates of enzymatic NADPH oxidation in the presence of quinones, nitroaromatics, aromatic *N*-oxides, or single-electron acceptors were determined using the value Δε_340_ = 6.2 mM^−1^cm^−1^, and they were corrected for the intrinsic NADPH-oxidase activity of *Bs*FNR (0.17 s^−1^) and/or nonenzymatic NADPH oxidation by high-potential quinones. When 50 µM of cytochrome *c* was added to the reaction mixture, its quinone- and nitroaromatic-mediated reduction was assessed according to Δε_550_ = 20 mM^−1^cm^−1^. The ferricyanide reduction rate was measured according to Δε_420_ = 1.03 mM^−1^cm^−1^. The rate of *Bs*FNR-catalyzed reduction of APADP^+^ by NADPH was determined according to Δε_363_ = 5.6 mM^−1^cm^−1^ [33]. APADPH was prepared in situ by reducing APADP^+^ with 10 mM of glucose 6-phosphate and 0.01 mg/mL of glucose 6-phosphate dehydrogenase, and its concentration was determined according to ε_365_ = 7.8 mM^−1^cm^−1^ [33]. NaCl was used to vary the ionic strength of the buffer solution. The stock solutions of organic compounds were prepared in DMSO; the final concentration of DMSO in reaction mixtures was 1% (*v*/*v*). The starting concentration for the oxidants ranged from 100 to 1000 µM, and every compound was measured in a series of measurements with 1.5× dilution for 6–10 different concentrations. Every measurement was performed thrice.

### 4.3. Presteady-State Kinetics

Pre-steady-state kinetics assays were performed under aerobic conditions using SX20 stopped-flow system (Applied Photophysics, Leatherhead, UK). The reduction of *Bs*FNR by NADPH and its reoxidation by a quinone or oxygen was evaluated at 460 nm and 600 nm. The reaction intermediate spectra were recorded at different wavelengths in the 450–750 nm range over various timepoints. The *Bs*FNR in syringe 1 (10 µM) was mixed with the contents of syringe 2 (100 µM of NADPH or 100 µM of NADPH and 66–500 µM of duroquinone). All measurements were performed in triplicate.

### 4.4. Photoreduction of BsFNR

The photoreduction of *Bs*FNR (20 µM) was performed under anaerobic conditions in 0.02 M Hepes/NaOH buffer solution (pH 7.0) using 5-deazaFMN (2 µM) and EDTA (8 mM) as photosensitizer and sacrificial reagent, respectively. The reaction mixture contained 10 mM of glucose, 50 nM of glucose oxidase, and 50 nM of catalase. Before the introduction of the concentrated enzyme stock solution, the solution in the sealed spectrophotometer cuvette was flushed with oxygen-free argon for 30 min. The 5-deazaFMN solution and cuvette contents were protected from light before illumination. The cell was subsequently illuminated for one-minute-long intervals using a 100 W incandescent lamp at a distance of 20 cm. The progress of the reaction was followed spectrophotometrically for 1–3 h; each spectrum is an average of two.

## Figures and Tables

**Table 1:** Steady-state rate constants of the reduction of non-physiological electron acceptors by 100 µM of NADPH catalyzed by *Bs*FNR. The $E_{7}^{1}$ values were taken from [37,38,39,40].

No.	Compound	$E_{7}^{1}$, V	$k_{\mathrm{cat}}^{\mathrm{app}}$, s^−1^	$k_{\mathrm{cat}}/K_{m}$, M^−1^s^−1^
Quinones				
1	1,4-Benzoquinone	0.090	209.8 ± 5.5	3.7 ± 0.4 × 10^6^
2	2-Methyl-1,4-benzoquinone	0.010	267.3 ± 7.8	2.3 ± 0.3 × 10^6^
3	2,3-Dichloro-1,4-naphthoquinone	−0.036	58.2 ± 1.1	3.5 ± 0.3 × 10^6^
4	2,3-Dimethyl-1,4-benzoquinone	−0.074	301.9 ± 19.8	1.1 ± 0.1 × 10^6^
5	2,6-Dimethyl-1,4-benzoquinone	−0.080	183.1 ± 5.0	1.0 ± 0.1 × 10^6^
6	5-Hydroxy-1,4-naphthoquinone	−0.090	221.4 ± 2.6	4.4 ± 0.2 × 10^6^
7	5,8-Hihydroxy-1,4-naphthoquinone	−0.110	96.0 ± 3.5	5.9 ± 0.4 × 10^6^
8	9,10-Phenanthrenequinone	−0.120	65.3 ± 2.1	7.5 ± 0.8 × 10^6^
9	1,4-Naphthoquinone	−0.150	180.1 ± 28.8	6.5 ± 0.5 × 10^5^
10	5-Hydroxy-2-methyl-1,4-naphthoquinone	−0.156	142.7 ± 8.7	6.0 ± 0.4 × 10^5^
11	2-Methyl-1,4-naphthoquinone	−0.200	68.9 ± 8.3	1.9 ± 0.3 × 10^5^
12	Tetramethyl-1,4-benzoquinone	−0.260	13.3 ± 1.2	9.9 ± 0.8 × 10^4^
13	9,10-Anthraquinone-2-sulphonate	−0.380	16.2 ± 0.6	6.0 ± 0.7 × 10^4^
14	2-Hydroxy-1,4-naphthoquinone	−0.410	2.5 ± 0.4	1.6 ± 0.3 × 10^4^
15	2-Hydroxy-3-methyl-1,4-naphthoquinone	−0.460	5.8 ± 0.4	1.5 ± 0.2 × 10^4^
**Nitroaromatic compounds**				
16	2,4,6-Trinitrophenyl-*N*-methylnitramine (tetryl)	−0.191	43.4 ± 6.0	6.0 ± 0.5 × 10^4^
17	*N*-methylpicramide	−0.225	17.5 ± 2.4	3.0 ± 0.2 × 10^4^
18	2,4,6-Trinitrotoluene (TNT)	−0.253	15.3 ± 0.6	4.0 ± 0.5 × 10^4^
19	Nitrofurantoin	−0.255	35.3 ± 7.2	1.8 ± 0.3 × 10^4^
20	Nifuroxim	−0.255	38.2 ± 4.0	4.4 ± 0.4 × 10^4^
21	*p*-Dinitrobenzene	−0.257	9.3 ± 0.5	2.3 ± 0.3 × 10^4^
22	*o*-Dinitrobenzene	−0.287	6.2 ± 0.4	1.4 ± 0.2 × 10^4^
23	2-Nitrobenzaldehyde	−0.308	26.4 ± 2.7	1.1 ± 0.2 × 10^4^
24	3,5-Dinitrobenzamide	−0.311	20.9 ± 1.3	1.4 ± 0.2 × 10^4^
25	4-Nitrobenzaldehyde	−0.325	20.8 ± 1.8	7.6 ± 0.7 × 10^3^
26	3,5-Dinitrobenzoic acid	−0.344	4.7 ± 0.3	2.5 ± 0.3 × 10^3^
27	*m*-Dinitrobenzene	−0.348	12.1 ± 0.7	6.1 ± 0.7 × 10^3^
28	4-Nitroacetophenone	−0.355	16.2 ± 1.6	5.7 ± 0.4 × 10^3^
29	5-(Aziridin-1-yl)-2,4-dinitrobenzamide (CB-1954)	−0.385	3.6 ± 0.4	4.3 ± 0.2 × 10^3^
30	2-Amino-4,6-dinitrotoluene	−0.417	2.0 ± 0.1	8.3 ± 0.9 × 10^3^
31	4-Amino-2,6-dinitrotoluene	−0.449	0.7 ± 0.04	4.3 ± 0.5 × 10^3^
32	3-Nitro-1,2,4-triazolone	−0.472	0.3 ± 0.03	7.3 ± 0.9 × 10^2^
33	4-Nitrobenzyl alcohol	−0.475	1.4 ± 0.1	8.2 ± 0.9 × 10^2^
**Aromatic *N*-oxides**				
34	7-Trifluoromethoxytirapazamine	−0.345	5.4 ± 0.3	1.2 ± 0.1 × 10^4^
35	7-Fluorotirapazamine	−0.400	6.2 ± 0.2	1.2 ± 0.1 × 10^4^
36	Tirapazamine	−0.456	5.6 ± 0.3	3.5 ± 0.4 × 10^3^
37	7-Ethoxytirapazamine	−0.494	2.3 ± 0.4	5.8 ± 0.5 × 10^3^
**Single-electron acceptors**				
38	Ferricyanide ^a^	0.410	557.5 ± 18.1	6.3 ± 0.8 × 10^6^
39	FeEDTA^−^	0.120	2.0 ± 0.1	5.7 ± 0.6 × 10^3^
40	Benzyl viologen	−0.354	38.4 ± 3.7	1.9 ± 0.3 × 10^5^

^a^ the $k_{\mathrm{cat}}^{\mathrm{app}}$ and $k_{\mathrm{cat}}/K_{m}$ values were calculated according to the reduction of ferricyanide on a single-electron basis.

## Data Availability

The data can be provided by the authors upon reasonable request.
